# Sensitive determination of hydrogen peroxide in real water samples by high spin peroxo complex

**DOI:** 10.3906/kim-1909-10

**Published:** 2020-04-01

**Authors:** Tuğba YAVUZ, Levent PELİT

**Affiliations:** 1 Department of Chemistry, Faculty of Science, Ege University, Bornova, İzmir Turkey

**Keywords:** Hydrogen peroxide determination, high spin peroxo complex, spectrophotometry, real water samples

## Abstract

In this paper, a fast, cheap, simple, sensitive and selective spectrophotometric method based on high spin peroxo-Fe(III)-EDTA complex in the alkaline medium was developed for the determination of hydrogen peroxide (H_2_O_2_) in real water samples. The purple-coloured complex with a maximum absorbance at a wavelength of 525 nm was formed. Various parameters such as type of stabilizer reagent and its concentration, reaction time, Fe(III), EDTA and NH_3_ concentration were optimized. The method was confirmed with the Beer’s law with a molar absorption coefficient of 267.36 L mol^-1^ cm^-1^ in the 8.3 ×10^-6^ –4.08 ×10^-3^ mol/L concentration range. Sandell’s sensitivity of the proposed method was also calculated as 0.188 μg/cm^2^ . LOD and LOQ were determined as 2.5 ×10^-6^ and 8.3 ×10^-6^ mol/L, respectively. Intraday and interday relative standard deviation of the proposed method for 2.0 ×10^-4^ mol / L of H_2_O_2_ were found as 1.5% and 6.1%, respectively. The developed method is suitable for fast monitoring of H_2_O_2_ in different types of aqueous water samples without any sample preparation steps and acceptable recovery values between 90% and 118% were obtained. In the sample analysis, H_2_O_2_ removed solutions from the real water samples were used for blank correction in their analysis and this process provides more reliable and accurate results in real sample analysis.

## 1. Introduction

Hydrogen peroxide (H_2_O_2_) is a colorless and thermodynamically unstable liquid. It is used in several industrial processes such as textile, food, pharmaceutical and dental products and environmental protection where it acts as an oxidizing, cleaning and sterilizing agent due to its high oxidant power [1–5].

H_2_O_2_ can occur in real water samples as a result of an industrial application or natural reaction. Removal of hazardous substances in the field of water treatment is a well-known example for industrial applications [6,7]. On the other hand, the ways for the natural production of H_2_O_2_ in the environment are the photochemical reactions of dissolved organic matters [8], wet deposition [9], dry deposition [10,11] or biological reactions [12]. Hydrogen peroxide is a hazardous chemical to both human and environment in high concentration. For example, H_2_O_2_ can cause skin irritation, embolism, gastric irritation and respiratory arrest to human even at lower concentrations [13]. The overall conclusion of IARC was that H_2_O_2_ is not classified as a carcinogenic compound to humans but some mutagenic properties have been observed in in-vitro systems [14]. Therefore, there is still a controversion on the cancer making properties of H_2_O_2_ . H_2_O_2_ has also concentration dependent toxic effect on some organisms in aqueous ecosystems such as fish, microorganisms, zooplankton [15]. Moreover, OH radicals arising from hydrogen peroxide have close relations with photochemical reactions and redox reactions in water and play an important role in the ecological effects of other organic and inorganic chemical substances [16].

Effective monitoring and fast determination of H_2_O_2_ in real water samples are crucial analytical problems because of rapid degradation of it. Various analysis methods based on titrimetric [17,18], spectrophotometric [19–22], fluorometric [23–26], chemiluminescence [27] or electrochemical [28–30] techniques have been described for the determination of H_2_O_2_ . However, most of these methods do not have access to adequate sensitivity and they are time consuming. Therefore, we aimed to develop a spectrophotometric method to determine H_2_O_2_ in a rapid, reliable, and sensitive way.

As an unstable reagent, decomposition of H_2_O_2_ can also be catalysed by aqueous metal ions as Fe(II) and Fe(III) ions. This reaction is called as Fenton reaction [31] and the reaction between H_2_O_2_ and iron ions can occur in the presence of strong complex-forming agent such as ethylenediaminetetraacetic acid (EDTA) or diethylenetriaminepentaacetic acid (DTPA), at neutral or basic pH range. On the other hand, Fe(III) gives a low stable purple coloured high spin complex with H_2_O_2_ which was first published in 1956 [32] in basic solution in the presence of EDTA. The detailed spectroscopic analyses of this peroxo complex were studied and the reaction mechanism was proposed in the followed equations in literature [33].

[Fe(EDTA)H_2_O]^-^+H_2_O_2_ ⇄ [Fe^3+^ (EDTA)O_2_]^3-^+ 2H^+^+ H_2_O

[Fe(EDTA)H_2_O]^2-^+H_2_O_2_ ⇄ [Fe^3+^ (EDTA)O_2_ ]^3-^+ H^+^+ H_2_O

[Fe(EDTA)H_2_O]^-^+ HO_2_^-^ ⇄ [Fe^3+^ (EDTA)O_2_ ]^3-^+ H^+^+H_2_O

[Fe(EDTA)H_2_O]^-^+ HO^-^_2_ ⇄ [Fe^3+^ (EDTA)O_2_ ]^3-^+ H_2_O

In this work, we developed a very fast UV spectroscopic method for the determination of residual hydrogen peroxide in real water samples by utilizing high spin peroxo-iron(III)-EDTA complex as a colour agent in the presence of stabilizer reagent. The developed method is sensitive, rapid, simple, and reliable for the determination of H_2_O_2_ in real water samples. To the best of our knowledge, this is the first report on the selective determination of H_2_O_2_ in the aqueous sample by means of high spin peroxo complex.

## 2. Materials and method

### 2.1. Instrumentation

Spectrophotometric measurements were carried out by CARY 1OO Bio UV-visible double-beam spectrophotometer. UV absorption spectra were recorded at room temperature by Hellma Analytics high precision quartz cells (111-QS).

### 2.2. Reagents

All solutions were prepared in ultrapure water supplied from Millipore Milli Q system (18.2 MΩ). Solutions of EDTA, S_2_ O^2-^_3_, e(III), and NH_3_ were prepared from 99% (w/w) Na_2_ (H_2_ EDTA) (Merck), 99.9% (w/w) Na_2_ S_2_ O_3_ (Sigma), 99.9% (w/w) FeCl_3_ .6H_2_O (Merck), and 25% (w/w) NH_3_ (Merck), respectively. Stock solutions of interfering ions were prepared by dissolving suitable salt in water. All of the other reagents and solvents used were of analytical reagent grade.

H_2_O_2_ standard solutions were prepared daily by dilution of a 35% (w/w) stock solution of H_2_O_2_ (Merck). Stock solutions of H_2_O_2_ (0.1 mol/L) were freshly prepared and standardized by the iodometric method against standardized 0.1 mol/L Na_2_ S_2_ O_3_ solution. Na_2_ S_2_ O_3_ solution was also standardized by the same method against IO^-^_3_ primer standard.

### 2.3. Preparation of complexing reagent

Fe(III), EDTA, and NH_3_ containing complexing reagent was prepared in water. For this purpose, solid FeCl_3_. 6H_2_O (0.209 g) was transferred to a beaker and was dissolved in 10 mL of ultrapure water. Then solid Na_2_ H_2_ EDTA (5.2 g) was added to this mixture and was stirred until all compound was completely dissolved. After that 10 mL of 25% (w/w) NH_3_ stock solution was added to the mixture to get alkaline media. Finally, 0.204 g of solid Na_2_ S_2_ O_3_ was added to the mixture and was diluted to 25 mL in a volumetric flask.

### 2.4. Spectrophotometric analysis method for H_2_O_2_

All measurements were performed in a 3.5 mL quartz cell for the rapid measurement. The quartz cell was cleaned by 0.1 M hydrochloric acid and was rinsed with ultrapure water before analysis. Then cell was treated with acetone and then dried by pure nitrogen (99.9%) to remove residual water and acetone from inside of the cell. After that, 2.0 mL of H_2_O_2_ containing sample solution and 1.2 mL of concentrated NH_3_ were added to the quartz cell. Finally, 300 μL of complexing reagent (containing 0.5 mol/L EDTA, 0.03 mol/L Fe(III), 5 mol/L NH_3_, 0.5 mol/L S_2_ O^2-^_3_) was added to cell and the final concentration of the EDTA, Fe(III), NH_3_ and S_2_ O^2-^_3_ were 5.0 ×10^-2^ mol/L, 3.0 ×10^-3^ mol/L, 5.0 mol/L and 5.0 ×10^-2^ mol/L, respectively. The solution was mixed well for 2 s and the purple-coloured peroxo complex formed immediately. The spectrophotometric measurements were carried out at 525 nm in 5 s. Spectrophotometric measurements were recorded against suitable blank solution.

### 2.5. Sample analysis method

The water samples were filtrated by 0.25 μm PTFE filter to remove the particles from water samples before analysis. Then the sample analysis was carried out according to part 2.4. Water samples except seawater were directly analyzed with the proposed method. Because of the precipitation formation after the addition of complexing reagent, the seawater sample was diluted 5 times by ultra-pure water before analysis.

## 3. Results and discussion

### 3.1. Absorption spectra

**Figure 1:**
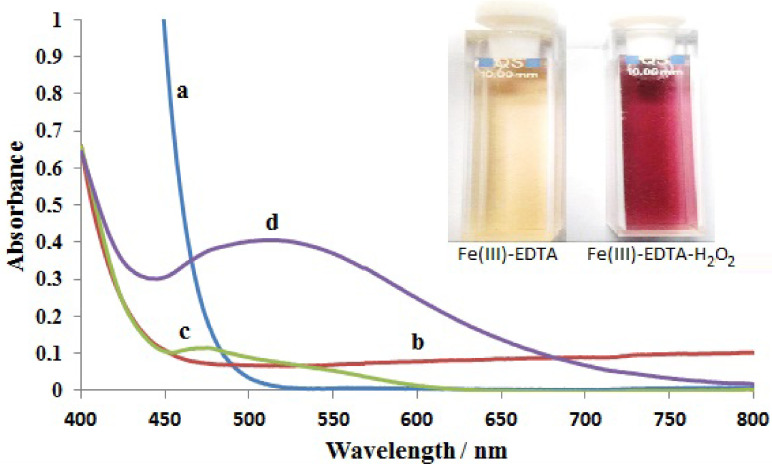
The spectra of a) Fe(III) at pH 2, b) Fe(III)-EDTA at pH 2, c) Fe(III)-EDTA in the presence of 5 M NH_3_, and d) Fe(III)-EDTA-H_2_O_2_ in the presence 5 M NH_3_ solutions (concentrations of Fe(III), EDTA, NH_3_, and H_2_O_2_ are 3.0 ×10^-3^ mol/L, 5.0 ×10^-2^ mol/L, 5 mol/L and 1,5 ×10^-2^ mol/L, respectively).

Absorption spectra of aqueous solutions of Fe(III) (a) and Fe(III)-EDTA (b) at pH 2 and Fe(III)-EDTA (c), and Fe(III)-EDTA-H_2_O_2_ (d) in the presence of 5 M NH_3_ were recorded between 800–400 nm without baseline correction. As can be seen from the figure, a very sharp charge transfer band was observed between 400 nm and 500 nm (Figure 1a) in acidic Fe(III) solution [34]. After addition of EDTA into the Fe(III) solution in the acidic medium, the sharp charge transfer band of the Fe(III) was shifted to shorter wavelengths (Figure 1b). A similar absorption spectrum was observed for Fe(III)-EDTA solution in the presence of NH_3_ (Figure 1c) and additional small absorption band was observed between 450 nm and 600 nm (λ_max_ 475 nm). This can be attributed to Fe(III)-EDTA complex formation in basic solution [35]. After addition of H_2_O_2_ to the Fe(III) - EDTA mixture, a purple-coloured complex was observed immediately at λ_max_ 525 nm in the presence of NH_3_ (Figure 1d). The colour of Fe(III) - EDTA and its peroxo complex were also presented in Figure 1 inset in the presence of NH_3_.

**Figure 2:**
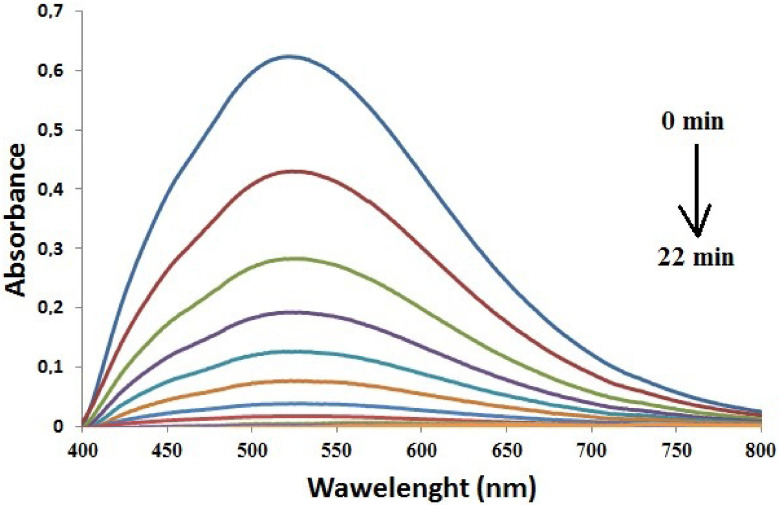
The change of absorbance of Fe(III)-EDTA peroxo complex against time. Measurements were monitored for 2 min interval time. Concentrations of Fe(III), EDTA, NH_3_ and H_2_O_2_ , 3.0 ×10^-3^ mol/L, 5.0 ×10^-2^ mol/L, 5 mol/L, 2 ×10^-3^ mol/L, respectively.

The peroxo complex was not stable and the color completely disappeared in 20 min for 2 ×10^-3^ mol/L concentration of H_2_O_2_ (Figure 2). Further experiments were carried out at 525 nm by photometrically by using baseline correction.

### 3.2. Optimization of the proposed method

Variety of parameters such as type of stabilizer reagent, reagent concentration, reaction time, and reagent addition order were optimized to get the most favourable conditions to attain maximum absorbance.

**Figure 3:**
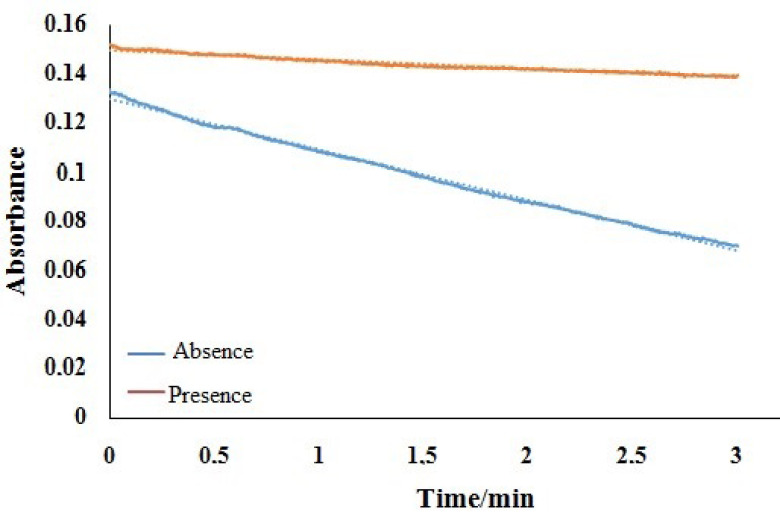
Absorbance change of peroxo complex against time in the absence and presence of Na_2_ S_2_ O_3_ (The concentration of Fe(III), EDTA, NH_3_ , H_2_O_2_, and Na_2_ S_2_ O_3_ are 0.002, 0.01, 0.5, 0.003, and 0.005 mol/L, respectively).

Very high absorbance decrease was observed because of the rapid decomposition of peroxo complex. Therefore, possible stabilizer reagents were tested for the stabilization of the complex’s absorbance in the presence of 5.0 ×10^-3^ mol/L stabilizer reagent and 3.0 ×10^-3^ M H_2_O_2_ concentration for 3 min. In the presence of CuSO_4_, BiNO_3_, Fe(NO_3_)_3_, MnSO_4_, MoO_3_, K_2_Cr_2_O_7_, KI, Na_2_ CO_3_ , Na_2_ B_4_O_7_, AgNO_3_ salt, the higher absorption decrease rates were observed. The absorption decrease rates of peroxo complex were the same in the presence and absence of Na_2_ C_2_O_4_ , HgCl_2_, (NH_4_)_2_Fe(SO_4_)_2_, SnO_2_, NaCl, and NaF salt. On the other hand, the absorption decrease rates of the peroxo complex decreased in presence of CoSO_4_, NiNO_3_, KCl, K_2_CrO_4_, Na_2_ SO_3_, and Na_2_ S_2_ O_3_ salts. The lowest absorption decrease rate was obtained in the presence of Na_2_ S_2_ O_3_ so this chemical was selected as stabilizer reagent for further experiments. The absorbance change and decrease percentage of peroxo complex against time in the absence and presence of Na_2_ S_2_ O_3_ are presented in Figure 3 and Table 1. As can be seen from Table 1, the absorbance of peroxo complex is nearly constant during the first 10 s after addition of complexing reagent in the presence of Na_2_ S_2_ O_3_. Therefore, the H_2_O_2_ analysis of samples was performed in 10 s after the addition of the complexing reagent.

**Table 1:** The percentage of absorbance decrease of Fe-EDTA-H_2_O_2_ complex.

Time (s)	Absorbance decrease (%) In the absence	Absorbance decrease (%) In the presence
10	4.2	1.0
15	5.6	1.5
20	7.8	2.0
30	11.2	2.4
60	18.4	4.0
90	26.4	5.7
120	34.0	6.3
180	39.0	6.9

The decomposition of the Fe (III)-EDTA-H_2_O_2_ complex can be attributed to the catalytic degradation of the complex by trace Fe(II) in the medium [31]. A small amount of Fe(II) forms in the medium by the Fe(III)/Fe(II) redox equilibrium and this Fe(II) catalyses the decomposition of the H_2_O_2_ to oxidation product O_2_ according to the following quasi reversible reaction.

2H_2_O_2_⇋+2H_2_O_2_

The stability effect of S_2_ O^2-^_3_ as a strong reducing compound can be attributed to the reduction of a part of forming O_2_ by S_2_ O^2-^_3_ according to quasi reverse reaction. Reformation of decomposed H_2_O_2_ decreases the decomposition rate of peroxo complex.

**Figure 4:**
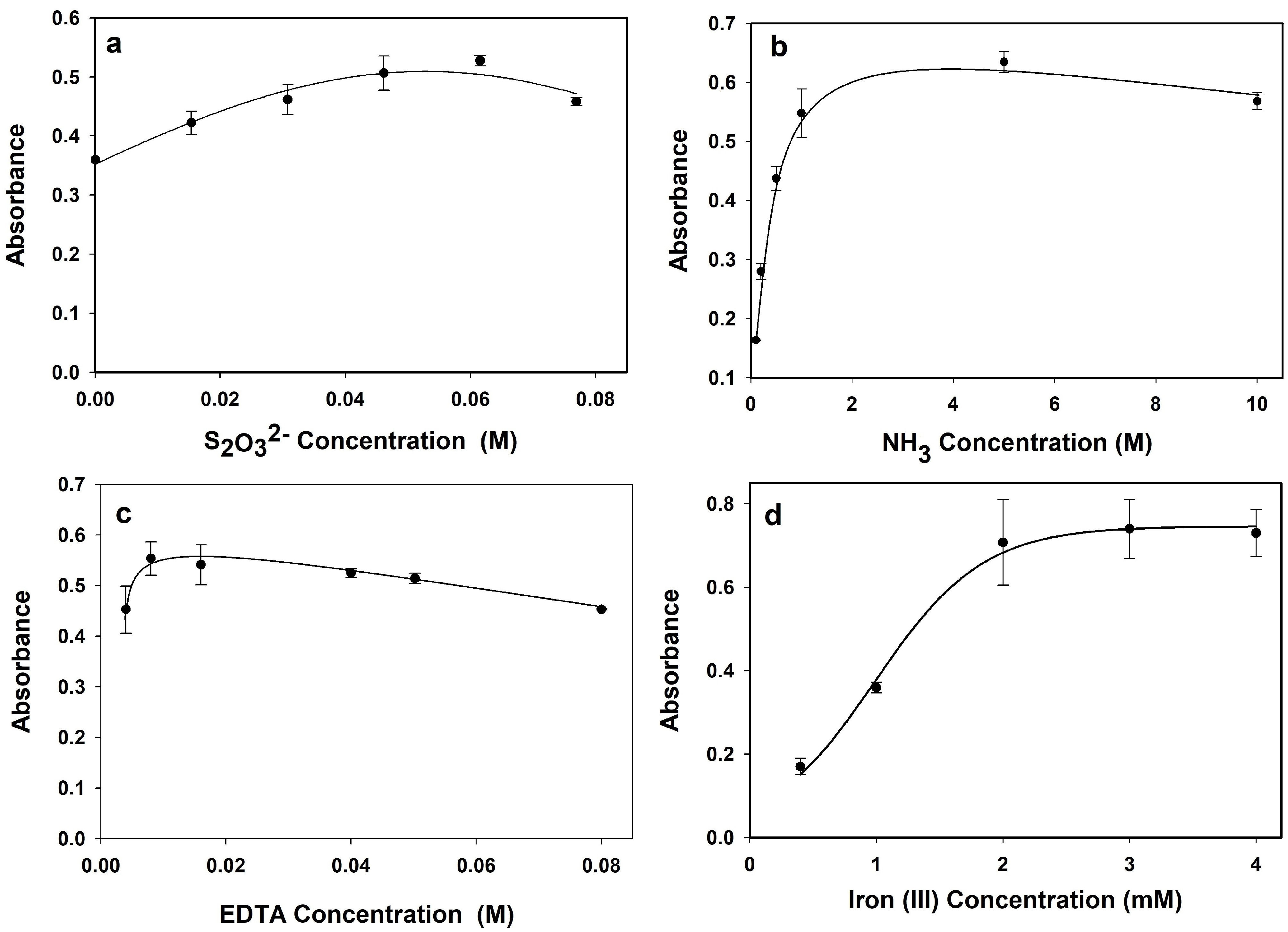
The effect of a) S_2_ O−2 3 concentration, b) NH_3_ concentration, c) EDTA concentration, and d) Fe(III) concentration.

The concentration of stabilizer reagent should be optimized to get more stable absorbance of peroxo complex. The effect of Na_2_ S_2_ O_3_ concentration was investigated up to 0.08 mol/L. As can be seen from the Figure 4a, the absorbance of peroxo complex was increased up to 0.06 mol/L Na_2_ S_2_ O_3_ concentration. A small absorbance decrease was observed after Na_2_ S_2_ O_3_ concentration was more than 0.06 mol/L. Therefore, further experiments were carried out in the presence of 0.06 mol/L of Na_2_ S_2_ O_3_ concentration.

The concentration of NH_3_ directly affects the absorption of peroxo complex. For this purpose, the effect of NH_3_ concentration was investigated in the range of 0.1 and 10 mol/L. As can be seen from the Figure 4b, the absorbance of peroxo complex increased by the addition of NH_3_ to 5.0 mol/L concentration. Ammonia concentrations of more than 5 M slightly reduced the absorbance (Figure 4b). Thus, further experiments were carried out in the presence of 5.0 mol/L NH_3_ concentration.

The concentration of EDTA is another important parameter and the effect of EDTA concentration on the absorbance of peroxo complex was investigated between 0.004 and 0.08 mol/L. As can be seen from the Figure 4c, there was no significant absorbance difference in the range of 0.004 and 0.05 mol/L EDTA concentration. After the concentration of EDTA exceeds 0.05 mol/L, a slight decrease was observed on the absorbance. For this purpose, further experiments were carried out in the presence of 0.05 mol/L EDTA concentration.

The concentration of Fe(III) directly affects the decomposition rate and also absorbance of the peroxo complex [36], so it should be optimized. The effect of Fe(III) concentration on the absorbance of peroxo complex was investigated between 0.0004 mol/L and 0.004 mol/L concentration range. Figure 4d shows that the absorbance of peroxo complex increased by the increasing of Fe(III) concentration up to 0.002 mol/L. Then no absorbance change was observed between 0.002 and 0.004 mol/L Fe(III) concentration. Decomposition percentages of the peroxo complex between 0.002 mol/L and 0.004 mol/L Fe(III) concentrations were also compared for 10 and 120 s and the results are summarized in Table 2. Decomposition percentage of peroxo complexes were similar in the first 10 s. However, 0.003 mol/L Fe(III) concentration showed the lowest absorption decrease for a longer period (Table 2). Therefore, optimum Fe(III) concentration was selected as 0.003 mol/L and optimum parameters are summarized in Table 3.

**Table 2:** The decrease of relative absorbance percentage of Fe-EDTA-H_2_O_2_ complex by Fe(III) concentration against time.

Time (s)	Relative absorbance decrease percentage of H_2_O_2_-Fe(III) complex (%)		
	0.002 mol/L Iron (III)	0.003 mol/L Iron (III)	0.004 mol/L Iron (III)
10	1.1	1.1	1.4
30	2.5	2.4	3.3
60	7.1	5.7	10.4
120	11.3	6.3	11.7

**Table 3:** The optimum parameters of the proposed method for the H_2_O_2_ determination.

Parameters	Optimum concentration mol/L
Fe(III) concentration	0.003
EDTA concentration	0.050
S_2_O^2-^_3_ concentration	0.050
NH_3_ concentration	5.00

### 3.3. Analytical merits of proposed method

**Figure 5:**
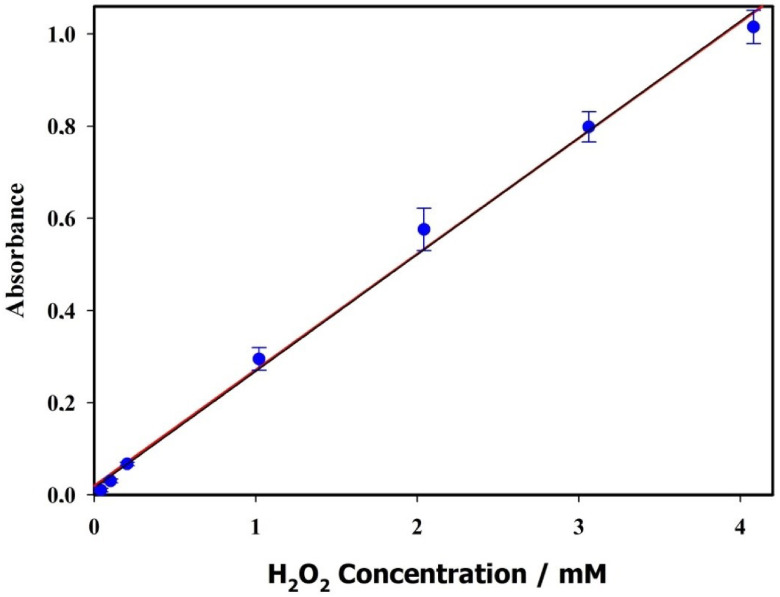
Calibration curve of the proposed method.

The obedience of absorbance values of peroxo complex against H_2_O_2_ concentrations to Beer’s law was investigated by varying the H_2_O_2_ concentration. A calibration curve was obtained by plotting absorbance of peroxo complex against H_2_O_2_ concentration up to 4.7 ×10^-3^ mol/L. Good obedience to Beer’s law is obtained in the range of 3.6 ×10^-6^ and 4.08 ×10^-3^ mol/L, and the calibration curve is presented in Figure 5.

Molar absorptivity coefficient of peroxo complex was calculated as 267.36 L mol^-1^ cm^-1^ under optimized conditions.

“The Sandell’s sensitivity is the concentration of the analyte (in μg mL−1) which will give an absorbance of 0.001 in a cell of path length of 1 cm and is expressed as μg cm−2 ” [37]. Sandell’s sensitivity of the peroxo complex was calculated as 0.188 μg/cm^2^ from the following equation:

S = εs.y

Where,

S = sandell’s sensitivity

εs = specific extinction coefficient

y = concentration of the substance in mg/L

The limit of detection (LOD) and limit of quantification (LOQ) for the proposed method were calculated according to the following equations:

LOD = 3 ×s/m

LOQ = 10 ×s/m

Where, s is the standard deviation of replicate measurement of blank signal under the optimized conditions and m is the slope of the calibration graph. LOD and LOQ were calculated as 2.5 ×10^-6^ mol/L and 8.5 ×10^-6^ mol/L, respectively.

In order to evaluate the intraday and interday precision of the proposed method, a solution containing 2.0 ×10^-4^ mol/L H_2_O_2_ was analysed in five replicates during the same day and 5 consecutive days. The interday and intraday percentage of relative standard deviations (RSD%) were found as 1.5% and 6.1%, respectively. The small values of the RSD% for intraday and interday indicate the high precision of the proposed method. The analytical figures of merits such as the molar absorptivity coefficient, limit of detection, and limit of quantitation of the proposed method are also summarized in Table 4.

**Table 4:** Analytical figure of merits of the proposed method for the determination of H_2_O_2_ .

Parameters	Value
Calibration equation	y = 267.36x + 0.0093
Linearity	8.5 ×10^-6^ and 4.08 ×10^-3^
R2	0.9984
Molar absorptivity coefficient	267 L mol^-1^ cm^-1^
Sandel’s sensitivity	0.188 (μg cm−2)
LOD	2.5 ×10^-6^ mol/L
LOQ	8.5 ×10^-6^ mol/L
Intraday RSD (for 2.0 ×10^-4^ mol/L)	1.5%
Interday RSD (for 2.0 ×10^-4^ mol/L)	6.1%

The performance of the proposed method for the determination of H_2_O_2_ in real water samples was compared with the other published spectrophotometric methods in literature (Table 5). When compared with the proposed method with other published methods, the compatible results were obtained.

**Table 5:** Comparison of the proposed method with other spectrophotometric techniques for the determination of H_2_O_2_.

Complexing reagent	LOD μmol/L	Linear range μmol/L	Sample type	Ref.
Osmium (VIII) and m-carboxyphenylfluorone	Not given	59–12,000	Not applied	[38]
Toluidine blue	1.41	0.2–14	Rain water	[39]
p-hydroxyphenylacetic acid (PHPA)	290	1.47–1470	Rain water	[40]
Titanium(IV)-XO	Not given	4–40	Water	[41]
Eriochrome black T	Not given	0.2–10	Not applied	[42]
Mo(VI)	Not given	50–400	Water	[43]
N,N-diethyl.p-phenylendiamine (DPD)	1.7	125–1000	Surface, tap water	[44]
Fe(III)-EDTA	2.5	8.3–4080	Drinking, tap, sea water	Proposed method

### 3.4. Interference studies

The effects of various well-known ions presented in the real water samples were studied in interference studies. The effect of interfering ions was investigated up to 5.0 ×10^-3^ mol/L concentration for the determination of 1.0 ×10^-4^ mol/L H_2_O_2_. An error of ±5% in the reading of absorbance was considered as tolerable concentration for H_2_O_2_ determination. The tolerance limits for various ions prevalent in real water samples are summarized in Table 6.

**Table 6:** Interfering ions for the H_2_O_2_ determination.

Cations					
Interfering Ion	Tolerance Limit (M)	Interfering Ion	Tolerance Limit (M)	Interfering Ion	Tolerance Limit (M)
NH+4	No Int	Cu2+	No Int	Bi3+	No Int.
Na+	No Int	Mn2+	No Int	Cr3+	No Int.
K+	No Int	Pb2+	No Int	As3+	No Int.
Ag+	No Int	Zn2+	No Int	Sn4+	No Int.
Mg2+	No Int	CO_2_+	No Int	Fe2+	1.0 ×10^-4^
Ca2+	No Int	Ni2+	No Int	Mo6+	1.0 ×10^-3^
Anions					
Interfering Ion		Interfering Ion	Tolerance Limit (M)	Interfering Ion	Tolerance Limit (M)
F−	Tolerance Limit (M)	NO−3	No Int	MoO_2_−4	No Int.
Cl−	No Int	NO−2	No Int	CO^2-^_3_	No Int.
Br−	No Int	SO−42	No Int	CrO−4	No Int.
I−	No Int	C2O−42	No Int	SO_2_−4	No Int.

No Int: No interference

As can be seen from Table 6, only Fe(II) shows a serious interfering effect on H_2_O_2_ determination. The interfering concentration for Fe(II) was found as 1.0 ×10^-4^ mol/L and this concentration is lower than the maximum Fe(II) concentration of real water samples. Therefore, this method can be applicable for H_2_O_2_ determination in real water samples without any further sample preparation steps.

### 3.5. Real sample applications

The proposed method was successfully applied to real water samples namely drinking water, tap water, and seawater. The blank solutions were directly prepared from each own sample. For this purpose, a suitable amount of complexing reagent except S_2_ O^-^_3^2^_ was added to real water samples and waited until the absorbance of peroxo complex totally disappeared. After the addition of stabilizer reagent to this solution, it was used as a blank solution for the background correction. This blank correction strategy provides a measure of a low level of H_2_O_2_ in complex real water samples. The precision of the proposed method was evaluated by the 3 replicate analysis of water samples in the presence of low, medium, and high level of H_2_O_2_ concentrations and the results are presented in Table 7. Two different commercial bottled waters (A and B), seawater, and tap water were analysed by the proposed method. The H_2_O_2_ concentrations of all samples were below the LOD value.

**Table 7:** H_2_O_2_ recovery values of the real water samples.

Recovery (%)					
Added concentration (M)	Found	Bottled water A	Bottled water B	Sea water	Tap water
1.0 ×10−5	<LOD	112 ±5	118 ±7	107 ±3	105 ±6
5.0 ×10−5	<LOD	97 ±4	91 ±8	90 ±5	107 ±8
1.0 ×10^-4^	<LOD	100 ±4	102 ±5	102 ±4	98 ±7

## 4. Conclusion

In conclusion, we have developed a fast spectrophotometric method for the determination of H_2_O_2_ by using coloured peroxo-iron(III)-EDTA complex in basic solutions. Absorbance stability of peroxo-Fe(III)-EDTA complex was enhanced by adding S_2_ O^2-^_3_ to medium as a stabilizer reagent. The developed method provided a sensitive, simple, rapid, and inexpensive way for the determination of H_2_O_2_ in aqueous samples. The method allowed detection of low concentrations of H_2_O_2_ in a wide range from 8.3 ×10^-6^ to 4.08 ×10^-3^ mol/L with high repeatability (RSD%: 1.6% for intraday, 6.5% for interday). The coloured complex formation between H_2_O_2_ and Fe(III)-EDTA is very sensitive and can be applied for the determination of H_2_O_2_ in real water samples without any further sample preparation step. The LOD value of the proposed method is suitable for the determination of H_2_O_2_ in real water samples with acceptable recovery values. The main advantage of the method is the use of blank solution directly from the targeted water sample and it provides high precision for detection of the low-level concentration of H_2_O_2_ even in complex matrices.
